# Supplementary material for: Leaf water potential of field crops estimated using NDVI in ground-based remote sensing—opportunities to increase prediction precision
Source: PeerJ. 2021 Aug 18;9:e12005. doi: 10.7717/peerj.12005 (PMC8380031; doi:10.7717/peerj.12005)
Supplement: Supplemental Information 9 — Time series plots of scanned NDVI values of the corn canopies under full and deficit irrigation regimes (blue open and red solid circles, respectively) at nine times on June 7, 2018. The NDVI values presented in each of the sub-figures represent the complete scans done at a particular hour across the northeast quarter of the center pivot field (see Fig. 3A in the main text), covering three full and three deficit plots. Also in each sub-figure, the average NDVI values for the full and deficit plots are indicated along with dotted lines (values from full irrigation are always higher than those from deficit irrigation). [file peerj-09-12005-s009.pdf]

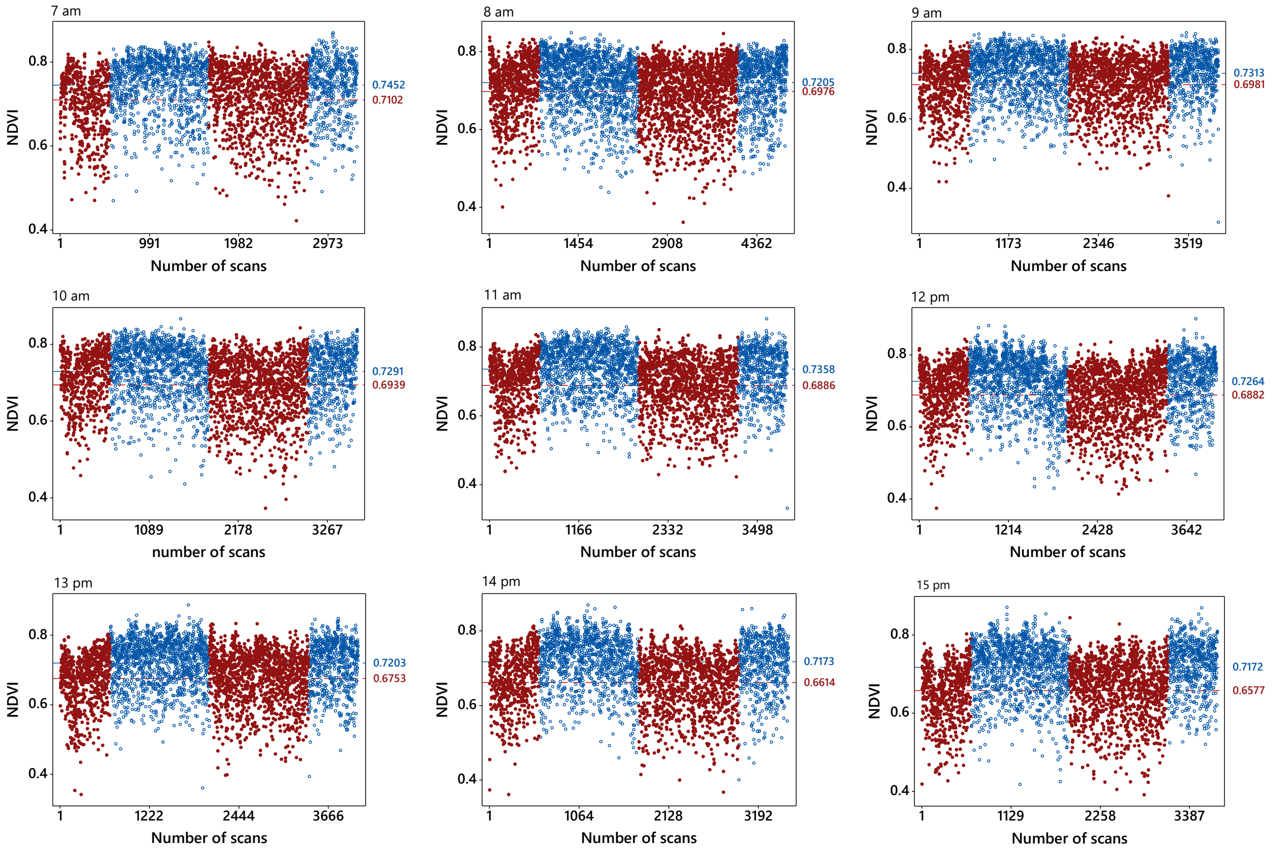

Figure S1: Time series plots of scanned NDVI values of the corn canopies under full and deficit irrigation regimes (blue open and red solid circles, respectively) at nine times on June 7, 2018. The NDVI values presented in each of the sub-figures represent the complete scans done at a particular hour across the northeast quarter of the center pivot field (see Fig. 3A), covering three full and three deficit plots. Also in each sub-figure, the average NDVI values for the full and deficit plots are indicated along with dotted lines (values from full irrigation are always higher than those from deficit irrigation).
