# Supplementary material for: Leaf water potential of field crops estimated using NDVI in ground-based remote sensing—opportunities to increase prediction precision
Source: PeerJ. 2021 Aug 18;9:e12005. doi: 10.7717/peerj.12005 (PMC8380031; doi:10.7717/peerj.12005)
Supplement: Supplemental Information 10 — (A) NDVI scans obtained at 8 am were substantially lower than those obtained from 10 am, 12 pm, 2 pm, 4 pm and 6 pm when the cart was moved passing the deficit and full plots. (B) NDVI scans obtained at 9 am were similar in magnitude when compared with data values obtained from 11 am, 1 pm, 3 pm and 5 pm when the cart was moved passing the full and deficit plots after one hour of rest. (C) NDVI scans obtained between 8 am and 10 am from the wheat plots, including both the measurements made when the cart was moving across the wheat plots and those when it was at rest (i.e., stationary). Note that at 8 am and 10 am, the order of scans was from deficit to full plots, while at 9 am it was the opposite (i.e., from full to deficit plots). The dashed blue line indicates the “running average” values of NDVI, which were obtained as the 7th average signals (A7) from a discrete wavelet transform (DWT) to the first 65536 sensor records using the computer program of Dong et al. (2008). A major strength of DWT for signal analysis is that it preserves localized subtle and abrupt variations, while also capturing the overall signal trends over time. The horizontal cyan line indicates the mean value of the 65536 NDVI records. This shows that, following the 8 am measurement, the NDVI values kept increasing during the first stationary period until 9 am. After that, the values became stabilized, as seen during the stationary period from 9 am to 10 am. To further visualize the contrasts in NDVI values before and after the sensor stabilization was reached, the 7th average signals (A7 from DWT) were sampled at every 100th point for both the resting periods from 8-9 am and 9-10 am in the order the values were recorded, resulting in 219 and 293 sampled pairs of NDVI-time index values, respectively. As can be seen in panel (D), the NDVI values during the stationary period from 8 am to 9 am were more strongly positively correlated with the index number (blue circles) than they were for the per [file peerj-09-12005-s010.pdf]

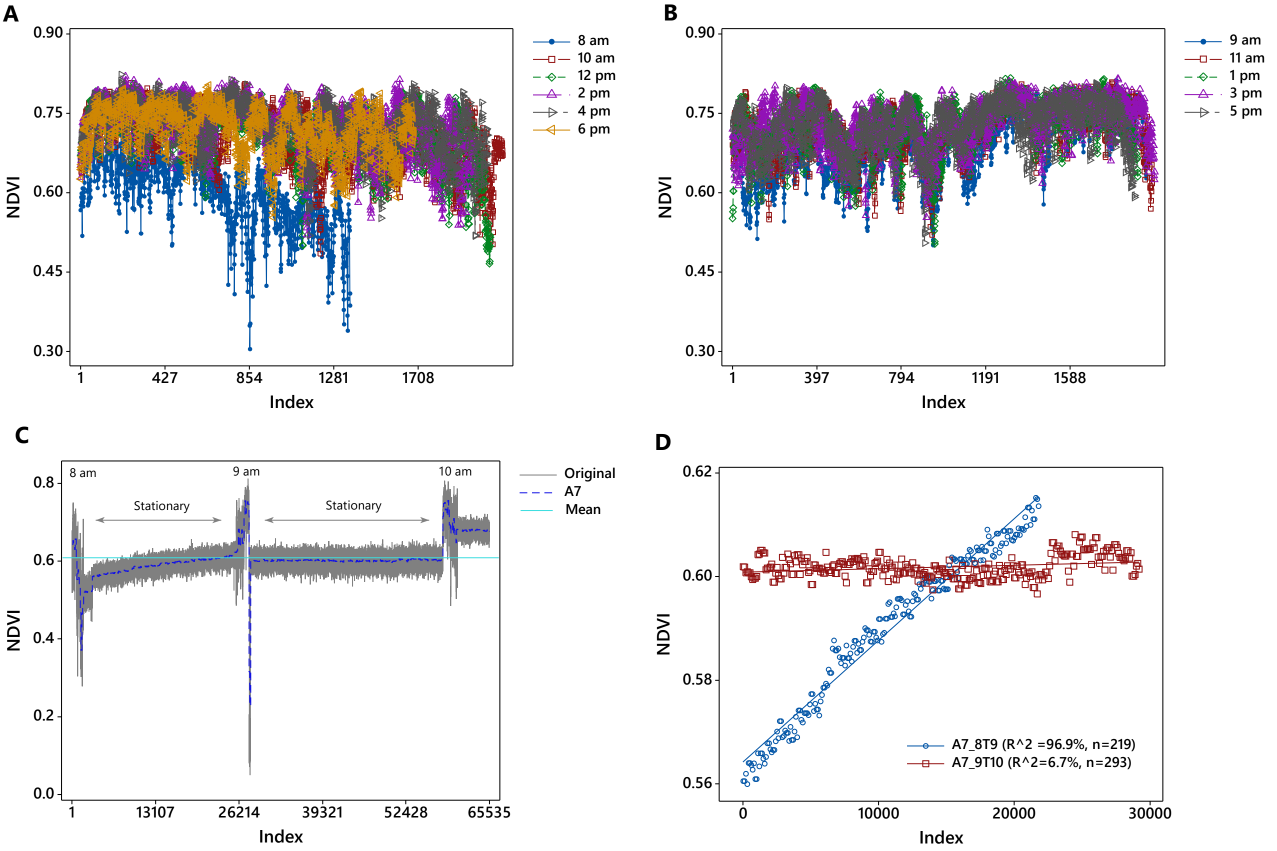

Figure S2: Justification for excluding the NDVI values measured from wheat plots at 8 am on April 12, 2018 from further analysis. (**A**) NDVI scans obtained at 8 am were substantially lower than those obtained from 10 am, 12 pm, 2 pm, 4 pm and 6 pm when the cart was moved passing the deficit and full plots. (**B**) NDVI scans obtained at 9 am were similar in magnitude when compared with data values obtained from 11 am, 1 pm, 3 pm and 5 pm when the cart was moved passing the full and deficit plots after one hour of rest. (**C**) NDVI scans obtained between 8 am and 10 am from the wheat plots, including both the measurements made when the cart was moving across the wheat plots and those when it was at rest (i.e., stationary). Note that at 8 am and 10 am, the order of scans was from deficit to full plots, while at 9 am it was the opposite (i.e., from full to deficit plots). The dashed blue line indicates the "running average" values of NDVI, which were obtained as the 7th average signals (A7) from a discrete wavelet transform (DWT) to the first 65536 sensor records using the computer program of Dong et al. (2008). A major strength of DWT for signal analysis is that it preserves localized subtle and abrupt variations, while also capturing the overall signal trends over time. The horizontal cyan line indicates the mean value of the 65536 NDVI records. This shows that, following the 8 am measurement, the NDVI values kept increasing during the first stationary period until 9 am. After that, the values became stabilized, as seen during the stationary period from 9 am to 10 am. To further visualize the contrasts in NDVI values before and after the sensor stabilization was reached, the 7th average signals (A7 from DWT) were sampled at every 100th point for both the resting periods from 8-9 am and 9-10 am in the order the values were recorded, resulting in 219 and 293 sampled pairs of NDVI-time index values, respectively. As can be seen in panel (**D**), the NDVI values during the stationary period from 8 am to 9 am were more strongly positively correlated with the index number (blue circles) than they were for the period after stabilization (from 9 to 10 am; see the red squares). Reference: *X. Dong, P. Nyren, B. Patton, A. Nyren, J. Richardson and T. Maresca, 2008. Wavelets for agriculture and biology: A tutorial with applications and outlook. BioScience 58: 445-453.*
