# Supplementary material for: Leaf water potential of field crops estimated using NDVI in ground-based remote sensing—opportunities to increase prediction precision
Source: PeerJ. 2021 Aug 18;9:e12005. doi: 10.7717/peerj.12005 (PMC8380031; doi:10.7717/peerj.12005)
Supplement: Supplemental Information 11 — Relationship between NDVI and leaf area index (LAI) for wheat, corn and cotton based on field measured values made on five days in 2018. Values of NDVI are averaged from field scans done at 10 am local time, and values for LAI are averaged from measurements made within 1–2 days from NDVI scans, except for values of corn at June 7 (93 DAP), which were interpolated linearly based on measured LAI values made on May 29 (84 DAP) and June 12, 2018 (98 DAP). For corn and cotton values from the full and deficit irrigation are indicated separately, while for wheat, the values from full and deficit irrigation are averaged, since the treatment was not replicated. Error bars indicate ± standard errors of means. [file peerj-09-12005-s011.pdf]

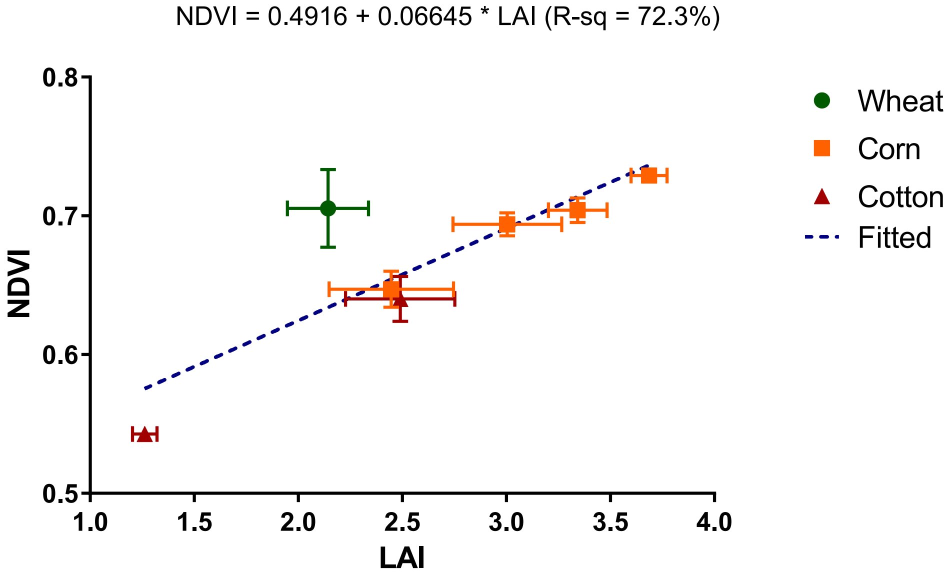

Figure S3: Relationship between NDVI and leaf area index (LAI) for wheat, corn and cotton based on field measured values made on five days in 2018. Values of NDVI are averaged from field scans done at 10 am local time, and values for LAI are averaged from measurements made within 1-2 days from NDVI scans, except for values of corn at June 7 (93 DAP), which were interpolated linearly based on measured LAI values made on May 29 (84 DAP) and June 12, 2018 (98 DAP). For corn and cotton values from the full and deficit irrigation are indicated separately, while for wheat, the values from full and deficit irrigation are averaged, since the treatment was not replicated. Error bars indicate $\pm$ standard errors of means.
