# Supplementary material for: Leaf water potential of field crops estimated using NDVI in ground-based remote sensing—opportunities to increase prediction precision
Source: PeerJ. 2021 Aug 18;9:e12005. doi: 10.7717/peerj.12005 (PMC8380031; doi:10.7717/peerj.12005)
Supplement: Supplemental Information 12 — Ranges of measured leaf osmotic potentials of ten cotton varieties planted in 2020 under irrigated and dryland conditions in Lytle (Irrigated) and Taylor (dryland), TX. Upper panel: dot plot for individual leaf samples. lower panel: dot plot for individual varieties. Reference of data source: X. Dong and D. A. Mott. (2021). Leaf osmotic potential and morphological traits of 43 cotton varieties growing in a rainfall gradient from southwest to central Texas. In: In Boyd, S., Huffman, M., Krogman, L., and Sarkissian, A., editors, Proceedings of the 2021 Beltwide Cotton Conferences. Pages 193-197, Virtual. National Cotton Council of America. [file peerj-09-12005-s012.pdf]

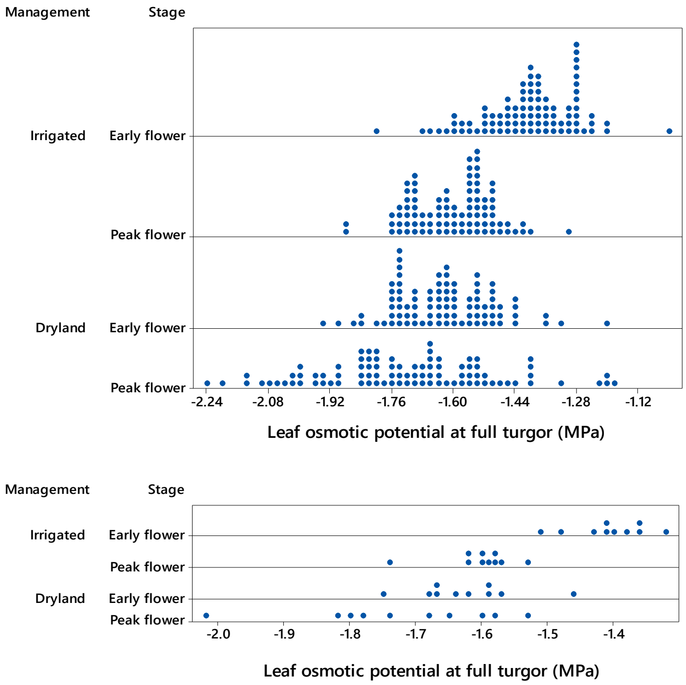

Figure S4. Ranges of measured leaf osmotic potentials of ten cotton varieties planted under irrigated and dryland condition in Lytle (irrigated) and Taylor (dryland), TX. Upper panel: dot plot for individual leaf samples. lower panel: dot plot for individual varieties. Source of data: X. Dong and D. A. Mott. (2021). Leaf osmotic potential and morphological traits of 43 cotton varieties growing in a rainfall gradient from southwest to central Texas. In: In Boyd, S., Huffman, M., Krogman, L., and Sarkissian, A., editors, *Proceedings of the 2021 Beltwide Cotton Conferences*. Pages 193-197, Virtual. National Cotton Council of America.
