# Supplementary material for: Leaf water potential of field crops estimated using NDVI in ground-based remote sensing—opportunities to increase prediction precision
Source: PeerJ. 2021 Aug 18;9:e12005. doi: 10.7717/peerj.12005 (PMC8380031; doi:10.7717/peerj.12005)
Supplement: Supplemental Information 13 — A comparison of the relationship between NDVI and leaf water potential (LWP) and that of the NDVI and air temperature (°C) for combined data of corn and cotton measured in 2018. [file peerj-09-12005-s013.pdf]

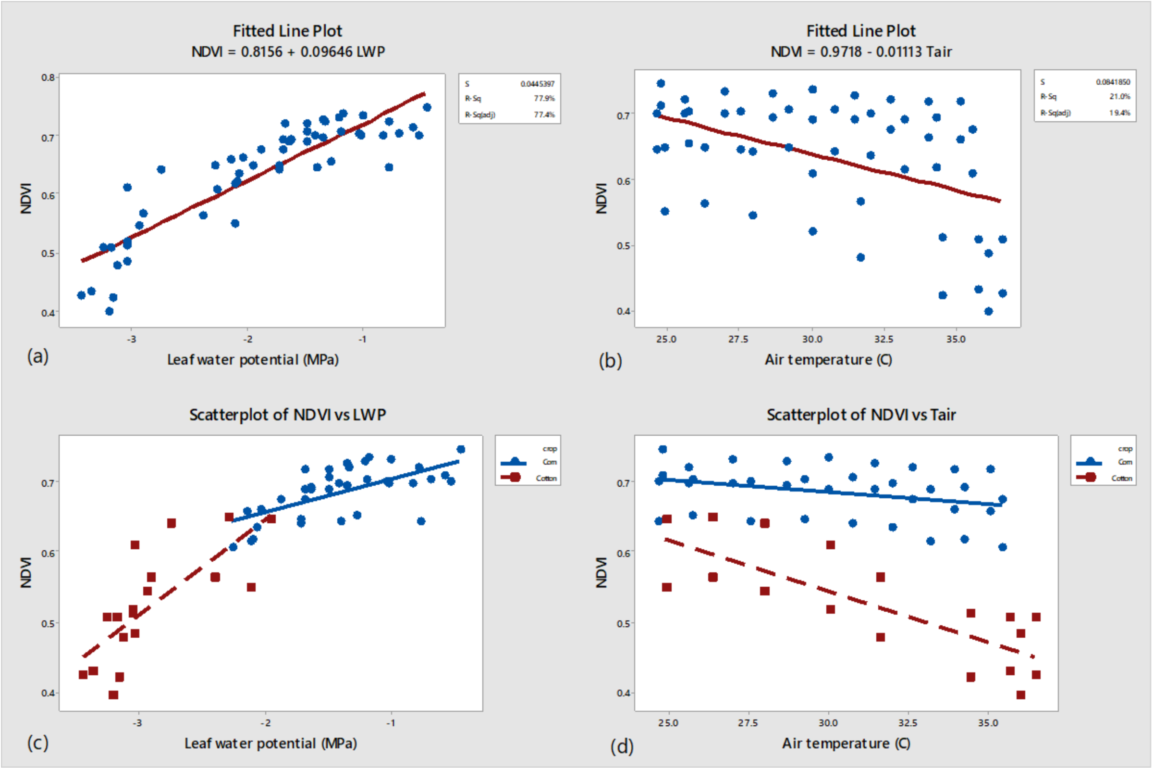

Figure S5. A comparison of the relationship between NDVI and leaf water potential (LWP) and that of the NDVI and air temperature (°C) for combined data of corn and cotton measured in 2018.
